# Burden and pattern of acute diarrhea in Thai children under 5 years of age: a 5-year descriptive analysis based on Thailand National Health Coverage (NHC) data

**DOI:** 10.1186/s12889-022-13598-8

**Published:** 2022-06-10

**Authors:** Busara Charoenwat, Kunanya Suwannaying, Watuhatai Paibool, Napat Laoaroon, Sumitr Sutra, Kaewjai Thepsuthammarat

**Affiliations:** 1 Department of Pediatrics, Srinagarind Hospital, Faculty of Medicine, Khon Kaen University, 123 Mitrapap road, Muang Khon Kaen, Khon Kaen, 40002 Thailand; 2 Clinical Epidemiology Unit, Srinagarind Hospital, Faculty of Medicine, Khon Kaen University, Khon Kaen, Thailand

**Keywords:** Acute diarrhea, Children under 5 years of age, Children

## Abstract

**Background:**

The incidence of acute diarrhea in Thai children under five years of age has increased over the last three decades. Even though mortality has significantly declined, the burden and cost of medical treatment are still high. Our objectives are to describe the burden and pattern of acute diarrhea cases that required admissions by Thai children under five years of age from 2015 to 2019.

**Methods:**

Data regarding the admission of acute diarrhea cases of Thai children with Thailand National Health Coverage (NHC) under five years of age from 2015 to 2019, recorded as International Statistical Classification of Diseases and Related Health Problems, tenth Revision, Thai Modification (ICD-10-TM), were analyzed.

**Results:**

The incidence trend of yearly acute diarrhea in children 0–5 years of age slightly increased from 33.36 cases per 1,000 population in 2010 to an average of 33.79 cases per 1,000 population/ year from 2015 to 2019 or approximately 0.43 cases per 1,000 population over the last decade while diarrhea-related mortality had a low, constant rate of 0.71 to 1.16 per 100,000 population per year. Two thirds of the mortality rate was observed in children under 1 year of age or 4.1 cases per 100,000 person-years in 5-year period (*P* < 0.01). The high cost of performing the medical treatment of approximately four hundred million baht per year. Seasonal variations demonstrated consistency with similar patterns during the cold and rainy seasons throughout the 5-year period. Regional distribution of the causative agent was also observed in Cholera, Typhoid, and Amoebiasis cases. A08: viral and other specified intestinal infections and A09: other gastroenteritis and colitis of infectious and unspecified origin were the two most common causes of diarrheal diseases.

**Conclusions:**

The incidence rate of acute diarrhea in Thai children under five years of age was higher while the mortality rate of acute diarrhea was lower than those in the past decade. A similar seasonal outbreak of acute diarrhea was seen during each examined year. The causative agent was not significant and was mainly unspecific.

**Trial registration:**

Number TCTR20220117002, date of registration: 17/01/2022, site: Thai Clinical Trials Registry, URL http://www.thaiclinicaltrials.org/show/TCTR20220117002

## Background

Diarrheal disease, which is defined as a decrease in the consistency of stool, loose stool and/or passing three or more stools in 24 hours [1, 2], remains the second largest cause of mortality in children, after pneumonia, globally, especially in developing countries [3]. The World Health Organization (WHO) and the United Nations Children's Fund (UNICEF) estimated that there are approximately 2 billion diarrheal episodes per year and approximately 1.9 million deaths of children under 5 years of age each year in 2013 and accounting for approximately 525,000 deaths in 2017 and 370,000 deaths in 2019 among children under age 5 worldwide [1, 4]. For diarrhea-related mortality, three-quarters of children are in African and Southeast Asian territories [3, 5].

A study by Sutra et al. [6] in the last two decade revealed the burden of diarrheal diseases and concluded that the incidence of diarrhea was higher than previously estimated while the mortality rate was lower. The Global Burden of Diseases, Injuries, and Risk factor Study 2015 (GBD 2015) [6] estimated that diarrheal disease was a crucial cause of mortality in all generations, particularly in children under 5 years of age (499,000 deaths, 95% UI 447,000–558,000). However, diarrheal-related mortality decreased by 20.8% (95% UI 15.4–26.1) from 2005 to 2015. The WHO has aimed to reduce diarrhea-related deaths in the under 5 years old population to below 1:1,000 live births by 2025 [7, 8].

The Bureau of Epidemiology of the Ministry of Public Health of Thailand revealed that the incidence of acute diarrhea in all age graudually decreased from 2013 to 2017 (17.6 cases per 1,000 population in 2013, 17.2 cases per 1,000 population in 2014, 16.9 cases per 1,000 population in 2015, 18.4 cases per 1,000 population in 2016, and 15.7 cases per 1,000 population in 2017, respectively) and the diarrheal-related mortality rate also declined. In 2013, there were 0.02 deaths per 100,000 population, 0.01 deaths per 100,000 population in 2014, 0.02 deaths per 100,000 population in 2015, 0.01 deaths per 100,000 population in 2016 and the same in 2017 [9]. For our country, Thai public health policy targets reducing the burden of diarrhea (based on admission rate, mortality, length of hospital stay (LOS), and cost of medical treatment) in children under 5 years old and reducing diarrheal-related death to less than 0.01 deaths per 100,000 population or to zero.

Over the past 30 years, the improvement of government health facilities services, socioeconomic status, sanitation, treated water supplies, personal hygiene, and oral rehydration salts (ORS), along with the promotion of exclusive breastfeeding, and, lastly, the rotavirus vaccine, were attributed to the improvement of diarrhea-related mortality outcomes in developing countries, and decreased diseases-related fatality in industrialized territories [3, 6, 10–12]. In Thailand, the universal health coverage (UCH) was realized in 2002 [13]. In 2010, the three major health schemes were the Universal Coverage Scheme (UC), the Civil Servant Medical Benefit Scheme (CSMBS) and the Social Security Scheme, which cover nearly all of the Thai population for the 23 diseases which cause the majority of the health burden in Thailand [14]. These health schemes was easily accessed and effective service system.

As a result of inadequate water, sanitation, and hand hygiene (WASH), both separately or in combination, they could be attributed to the burden of diarrheal disease and deaths in previous decades. The UCH also applied WASH interventions, as WHO’ s policies, to reduce the burden and deaths from diarrhea [15, 16]. The Thai government improved safe and sufficient drinking-water supply; effective household water treatment; qualified water storage; and systematically managed pipe water on permises. For sanitation practices, people gained access to the qualified sanitation facilities with sewery systems at both household and community level. Lastly, on the hand hygiene aspect, UCH launched a campaign to improved hand hygiene practices by encouraging handwashing with soap after latries use or before food preparation. This campaign has been open to the public since kindergarten.

The implication of accessibly UCH along with higher quality of socioeconomic status and health education showed significant reduction in diarrheal-related mortality rate in children under 5 years from 0.35 deaths per 100,000 population in 2001 to 0.10 deaths per 100,000 population in 2012 [5]. These interventions may have contributed to the better overall outcomes of acute diarrhea. The objectives of this study are to update data on the burdens (*i.e.*, admission rate, mortality, LOS, and cost of medical treatment), patterns, and geographic distribution of acute diarrhea in Thai children under 5 years of age by utilizing the most reliable data available from the Thailand National Health Coverage (NHC) data.

The burden of diarrheal disease is a pressing problem in Southeast Asia and Thailand, so it is important to understand the patterns and epidemiology of this burden. These analytic data will assist clinicians in understanding the etiology and outcome of diarrhea disease and in developing future national health schemes, prevention, and promotion policies.

## Methods

A 5-year descriptive analytic study on the data regarding the admission of acute diarrhea cases by Thai children under 5 years of age based on the UC scheme, which covered two thirds of Thai residents, from National Health Coverage (NHC) in a timeframe between the fiscal years of 2015 and 2019, was used in the study. The number of non-admission cases may be underestimated. For mild symptomatic diarrheal cases where they sought medical care from primary health care, a local hospital, or a private clinic, these medical records were unavailable or incomplete. The payment system for outpatient visits was per capita, so the data might be unreliable. Therefore, these datasets were excluded. The authors analyzed inpatient data that represented at least moderate severity of acute diarrhea that required admission. “Acute diarrhea” is characterized by a decrease in stool consistency, loose or liquid stool texture and/or an increase in the frequency of bowel movements to three or more in 1 day. The duration of the disease is classified as acute onset, lasting less than 7 days; prolonged diarrhea, lasting 8 to 13 days; and chronic or persistent diarrhea, lasting 14 days or more [17]. Information was extracted from the summary discharge of all hospitals in Thailand based on NHC data by using the International Statistical Classification of Diseases and Related Health Problems, tenth Revision, Thai Modification (ICD-10-TM). Acute diarrheal diseases are defined by ICD-10-TM as intestinal infectious disease, A00-A09. The code information was analyzed for the number of diarrheal-related admissions, LOS, and the rate of diarrheal-related deaths using the same ICD-10-TM code. Based on these coding, two categories were made. First, the nonspecific diagnosis of infectious disease, and second, the more specific diagnosis of infectious diseases. A04: other bacterial intestinal infections; A05: other bacterial foodborne intoxications, not elsewhere classified; A08: viral and other specified intestinal infections; and A09: other gastroenteritis and colitis of infectious and unspecified origin were included in the first category. A00: Cholera, A01: Typhoid, A02: other Salmonella infections, A03: Shigellosis, A06: Amoebiasis, and A07: other protozoal intestinal diseases were included in the second category. A02 and A03 were subcategorized to be defined as dysenteric diarrhea. The main objective was to determine the burden of the disease, which was based on admission rate, mortality, LOS, and cost of medical treatment. The minor objectives were the monthly admission rate, which was assessed to reveal the variation according to the climate of each season, and the three common specific diagnostic diseases in each province, which were presumed to be regional distributors. Data on basic demographics (age and gender), admission details, hospital region, LOS, mortality rate, and hospital expenditure were collected. The majority of the information was based on acute-onset diarrheal disease data; however, a number of cases of prolonged and persistent diarrhea were included to analyze the mortality rate, LOS, and health care expenditure. The denominator used in the calculation of diarrheal-related admission and diarrhea-related mortality rates was based on the UC scheme, which data covered two thirds of Thai residents. The diarrheal-related admission rate was calculated as per 1,000 population of the children aged 5 years and under while diarrhea-related mortality rate was calculated as per 1000,000 population of the same age groups. The estimated hospital expenditure of diarrheal-related admissions was calculated as cost per each year.

The present study was approved by the international review board, Center for Ethics in Human Research, Khon Kaen University, Human Research Ethics Committee (#HE 641,527). Consent was waived by Center for Ethics in Human Research, Khon Kaen University because of no personally identifiable data.

### Statistical methods

Continuous and categorical variables are described as medians (interquartile range), range (minimum and maximum), and frequencies (%). Diarrheal-related admission rate was analyzed per 1,000 population of the children aged 5 years and under and mortality rate are analyzed per 100,000 population for same age groups. The age groups were divided into 0–1 year, > 1 to 2 years, > 2 to 3 years, > 3 to 4 years, and > 4 to 5 years of age, and in each year. This rate is calculated by the Poisson regression test. The monthly incidence rate in each year is presented as seasonal peaks. The highest incidence rate of each causative pathogen per province is represented according to the regional distribution. A *P* value < 0.05 is considered statistically significant. The data are analyzed using the Stata software package, version 10 (StataCrop LP) program (Texas, USA).

## Results

**Fig. 1:**
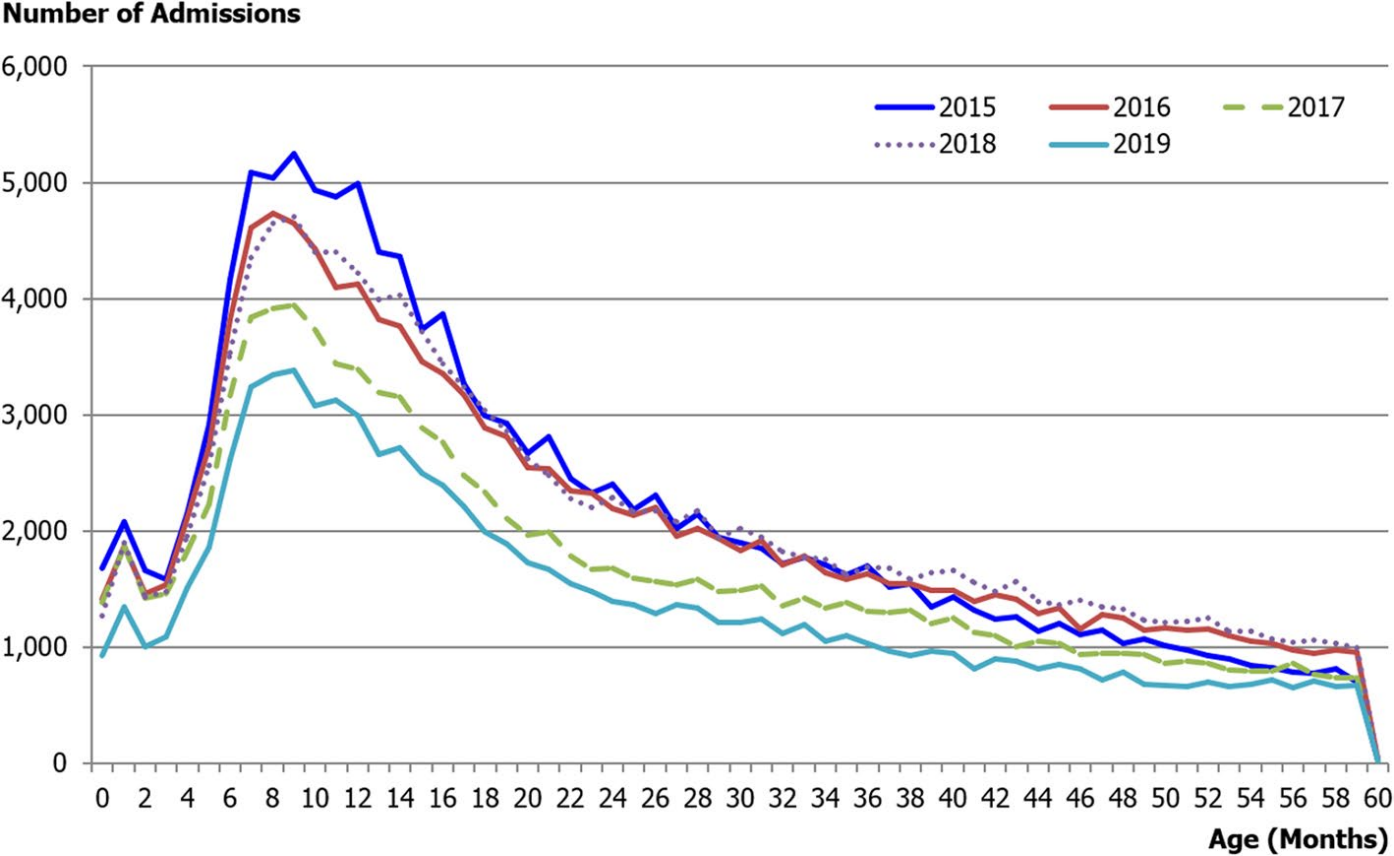
Acute diarrheal-related admissions in Thai children under 5 years by age (month) in fiscal year 2015 to 2019

For the 5-year study period, a total of 579,674 acute diarrheal-related admissions, in-patient department (IPD), of Thai children under 5 years of age were recorded, There were 28.92, 35.41, 30.92, 39.83, and 32.66 cases per 1,000 population in 2015, 2016, 2017, 2018, and 2019, respectively (Table 1). The mean diarrheal-related admissions rate was 33.79 cases per 1,000 population/ year from 2015 to 2019. Two-thirds of the IPD patients within 5 years period were infants and toddlers; there were 57.3 cases per 1,000 person-years (30.2%) and 51.6 cases per 1,000 person-years (29.5%) in children under 1 year of age and aged between 1 and less than 2 years, respectively, and the admission rate declined with increasing age (Table 2 and Fig. 1). Due to the limitation of causative confirmation tests, most diagnoses were based on clinical symptoms. The two most common causes relating to admission were A09: other gastroenteritis and colitis of infectious and unspecified origin; (83.0%) and A08: viral and other specified intestinal infections; (9.8%). Other specific diseases, including A00: Cholera, A01: Typhoid, A02: other Salmonella infections, A03: Shigellosis and A07: other protozoal intestinal diseases, were seen to have relatively lower rates: 0.01%, 0.2%, 0.6%, and 0.05%, respectively.

**Table 1:** Number of diarrheal-related admissions rate of Thai children under 5 years in fiscal year 2015–2019

Fiscal year	Diarrheal-related admissions rate(per 1,000 population)
2015	28.92
2016	35.41
2017	30.92
2018	39.83
2019	32.66

**Table 2:** Admission and mortality rates by age group of Thai children under 5 years in fiscal year 2015–2019

Age group (years)	Admission rate		Mortality rate		
	No (%)	(/1,000 person-years)	No (%)	(/ 100,000 person-years)	*P-value
< 1	174,799 (30.2)	57.3	126 (64.9)	4.1	< 0.001
1 to < 2	171,221 (29.5)	51.6	34 (17.5)	1.0	
2 to < 3	102,941 (17.6)	29.8	14 (7.3)	0.4	
3 to < 4	75,390 (13)	20.9	11 (5.7)	0.3	
4 to ≤ 5	55,323 (9.5)	14.8	9 (4.6)	0.2	

^*^P-value < 0.001 represented statistically significant mortality rate in in children aged less than 1 year

### Diarrheal-related mortality

Diarrheal-related mortality rate was significantly higher in children aged 1 year or less. In this age group, 64.9% (126/194) of all deaths or 4.1 deaths per 100,000 person-years occurred in first-year children (*P* < 0.001) in the 5-year period (Table 2). The number of deaths ranged from 31 to 47 deaths/year. The highest mortality cause was associated with A09: other gastroenteritis and colitis of infectious and unspecified origin, which comprised 81.4–96.8% of all deaths. (Table 3). Therefore, the diarrheal-related mortality rate ranged from 0.71 to 1.16 per 100,000 population per year (Table 4).

**Table 3:** The number of diarrheal-related mortality by etiology in Thai children under 5 years in fiscal year 2015–2019

Etiology (ICD-10TM)	Fiscal year, N (%)				
	2015	2016	2017	2018	2019
A02 Other Salmonella infections	3 (6.9)	1(3.2)	1 (2.8)	3 (6.4)	0 (0)
A03 Shigellosis	2 (4.7)	0 (0)	0 (0)	1(2.1)	0 (0)
A04 Other bacterial intestinal infections	1 (2.3)	0 (0)	2 (5.6)	1(2.1)	1 (2.7)
A06 Amoebiasis	0 (0)	0 (0)	0 (0)	0 (0)	1 (2.7)
A08 Viral and other specified intestinal infections	2 (4.7)	0 (0)	2 (5.6)	1(2.1)	3 (8.1)
A09 Other gastroenteritis and colitis of infectious and unspecified origin	35 (81.4)	30 (96.8)	31(86.1)	41(87.2)	32 (86.5)
Total	43	31	36	47	37

**Table 4:** The number of diarrheal-related mortality rate by LOS in fiscal year 2015–2019

Fiscal Year	LOS (Days)	Number of Admissions (%)	Number of deaths	Mortality rate (per 100,000 population)
2015	< 14	106,066(99.8%)	39	0.97
	> 14	182 (0.2%)	4	
2016	< 14	125,948 (99.8%)	27	0.71
	> 14	196 (0.2%)	4	
2017	< 14	105,716 (99.8%)	34	0.85
	> 14	193 (0.2%)	2	
2018	< 14	131,704 (99.8%)	44	1.16
	> 14	201 (0.1%)	3	
2019	< 14	109,309 (99.9%)	37	0.95
	> 14	159 (0.1%)	0	

### Diarrheal-related LOS and healthcare expenditure

Nearly all (99.8%) admissions involved a stay in the hospital less than 14 days, meaning that the diarrhea was solved within this period and not turned into chronic form. The diarrheal-related mortality rate in this group was smaller when compared to the group with an LOS of more than 14 days (Table 4).

The median estimated healthcare of diarrheal-related admissions was 443,782,932 Thai baht/year (interquartile range 396,958,609.5–512,391,546 Thai baht/year) or 13,266,656.7 United States dollar (USD)/year, (interquartile range 11,866,868.2–15,333,818 USD/year) (March 30, 2022, US Federal Statistical Release year). which the highest rate was found in 2018 (Table 5)**.**

**Table 5:** The healthcare expenditure of diarrheal-related admissions in fiscal year 2015–2019

Fiscal year	Healthcare expenditure (Thai baht)
2015	370,992,747
2016	492,806,412
2017	422,924,472
2018	531,976,680
2019	443,782,932

### Year-round variation

**Fig. 2:**
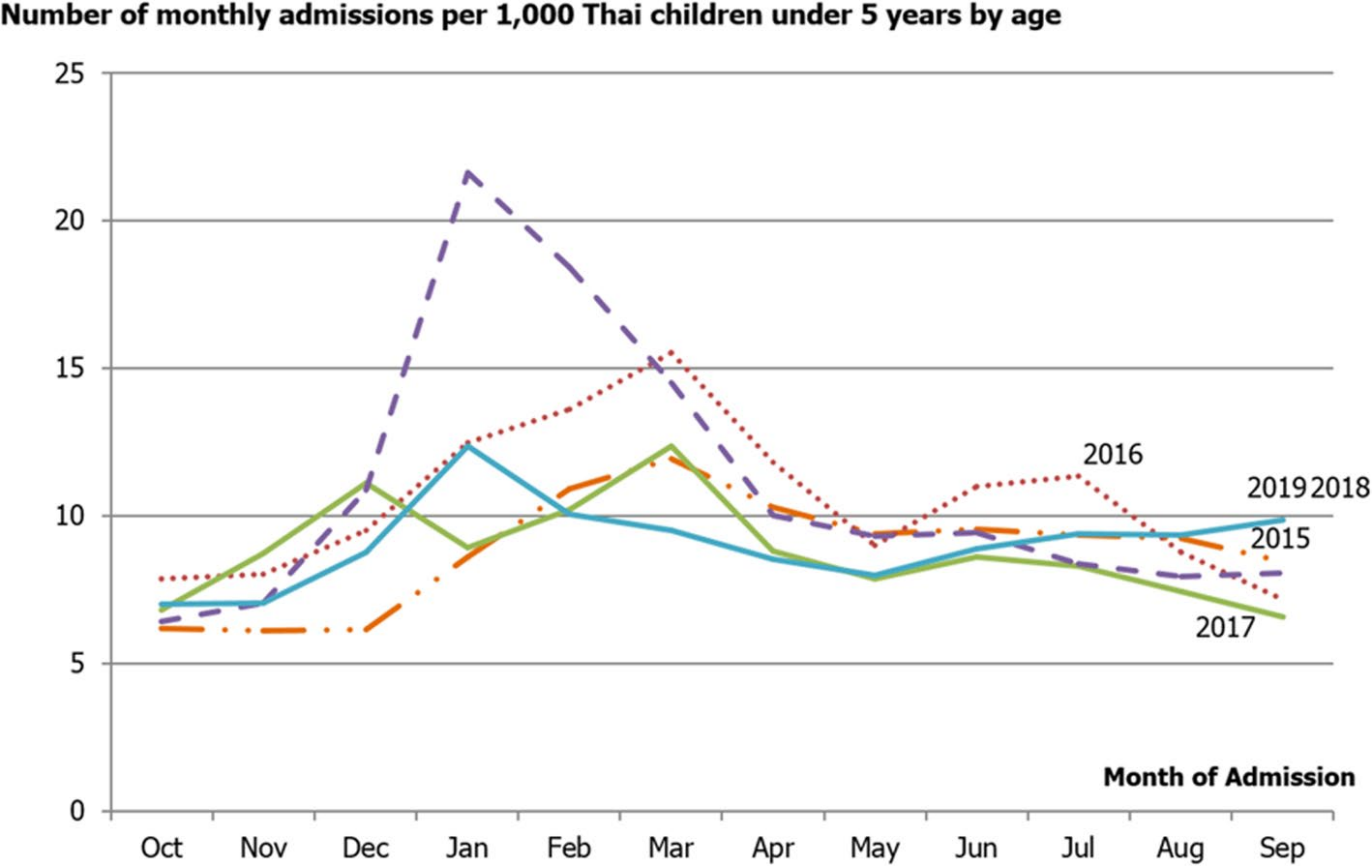
The number of monthly admissions per 1,000 Thai children under 5 years by age

According to the monthly incidences of diarrheal-related admissions, the bimodal peaks were consistent with this pattern over the 5 years (Fig. 2). The first high peak was observed in the cool climate; it sharply increased in November, reached a maximum in January, and then gradually decreased in February. The final low peak occurred during the early rainy season between May and July. In other months, a lower incidence of admissions was seen all year-round.

### Geographic distribution

**Fig. 3:**
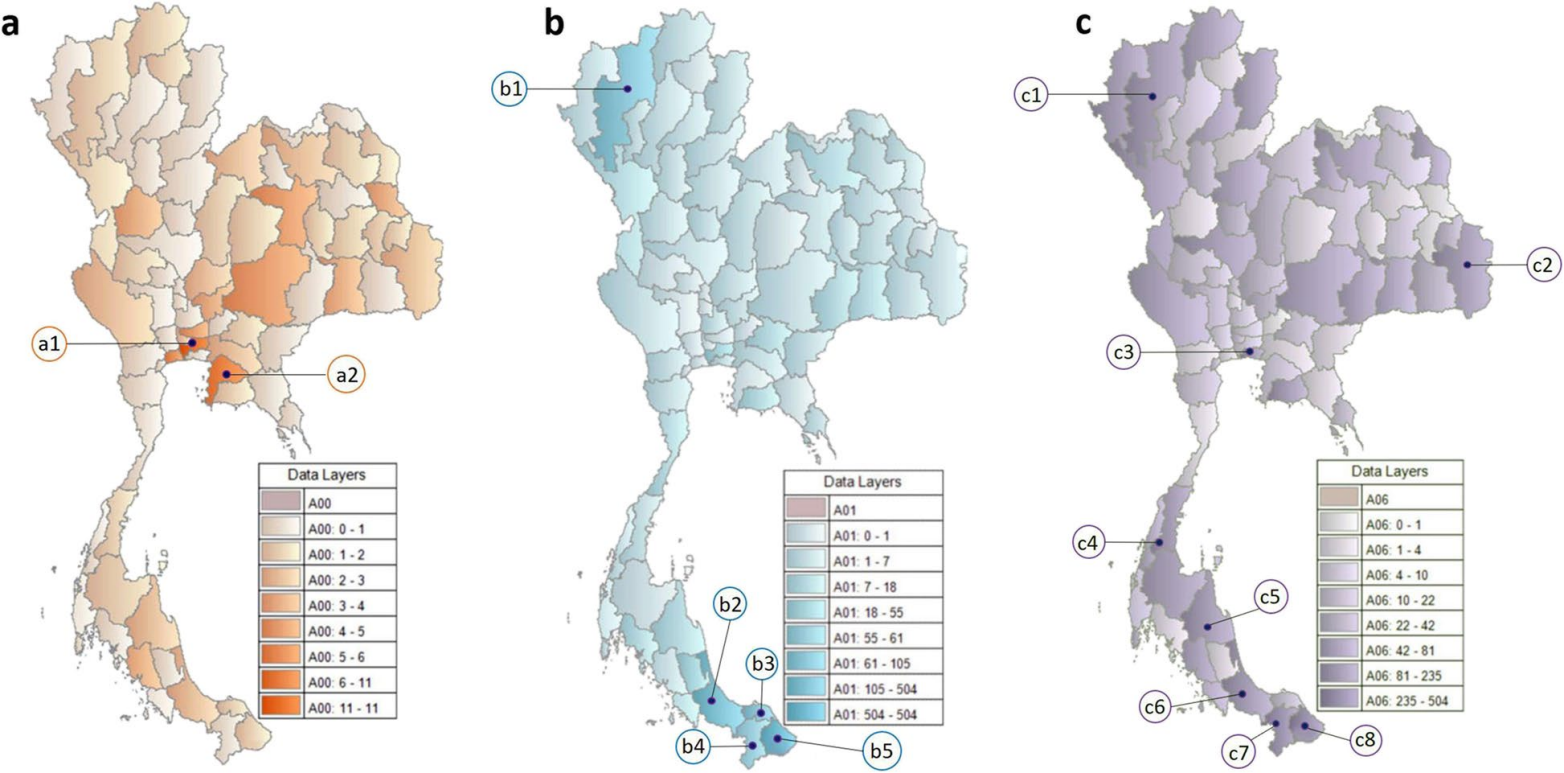
Geographic distribution of the three specific common foodborne pathogens: **a** Regional distribution of A00: Cholera; a1, Bangkok; a2, Chonburi, **b**) Regional distribution of A01: Typhoid; b1, Chiang Mai; b2, Songkhla; b3, Pattani; b4, Yala; b5, Narathiwat; and **c**) Regional distribution of A06: Amoebiasis; c1, Chiang Mai; c2, Ubon Ratchathani; c3, Bangkok; c4, Chumphon; c5, Nakhon sri thammarat; c6, Songkhla; c7, Yala; c8, Narathiwat, the gradient color bar in the right lower part of each picture indicates the density of the pathogen in each province

The three specific common foodborne pathogens, Cholera, Typhoid, and Amoebiasis, had distinctive regional distributions. Cholera, which causes severe watery diarrhea, had a high prevalence in the Bangkok metropolis of central and Chonburi Province in the eastern region. Chonburi Province is a coastal area with brackish water, which is a great habitat for *Vibrio* *cholera.* The incidence of Typhoid was highest in the southernmost part of the country (Songkhla, and Pattani, Yala, and Narathiwat provinces) and small portions scattered in the northeast (Chiang Mai province) For, Amoebiasis also occurred in the southern region (Chumphon, Nakhon sri thammarat, Songkhla, Yala, and, Narathiwat provinces) but has also seen a small number of cases in northeast (Chiang Mai province), eastern (Bangkok province), and southeast (Ubon Ratchathani province) regions (Fig. 3).

## Discussion

Diarrhea is a major global burden disease among children, largely in the under-5-year-old group. According to WHO and UNICEF in 2013, there are approximately 2 billion cases of diarrheal disease globally in each year, and 1.9 million diarrheal-related deaths in the under five years old group, and accounting for approximately 525,000 deaths in 2017and 370,000 deaths in 2019 among children under age 5 worldwide. [1, 4] as the second leading cause of mortality [3].

In Thailand, national health insurance consisted of three main schemes. Since 2002, the UC scheme was implemented, which covers two thirds of the Thai population. [5, 13, 18] Hospital-based data gathered from the Bureau of Epidemiology of the Ministry of Public Health of Thailand base on UC scheme from 2015 to 2019 were analyzed to estimate the burden and pattern of acute diarrhea in under 5 years old Thai children. Therefore, these data covered most Thai citizens. During the 5-year study period, the mean diarrheal-related admissions rate was 33.79 cases per 1,000 population. Notably, there was a striking peak in the admissions rate in 2018; 39.83 cases per 1,000 population In that time period from January to March, an outbreak of rotavirus diarrhea was detected. That was the reason why a high diarrheal-related admissions rate was observed.

Over the last 4 decades, the annual incidence of diarrhea in Thai children under 5 years of age had been changed over the time from 4 cases per 1,000 population in 1978 to 100 cases per 1,000 population in 1983 [19], which gradually decreased to 71.4 cases per 1,000 population in 2000 and sharply decreased to 33.36 cases per 1,000 population in 2010. [5] In 2015, GBD 2015 assessed that diarrheal episodes from 2005 to 2015 in Thai children younger than 5 years decreased by half (-47.3%, (-59.1 to – 32.9)) and episodes of children younger than 5 years declined by 10.4% (9.1–11.6%) worldwide. [6] This study also revealed a slightly increasing in diarrheal-related admissions rate by approximately 0.43 cases per 1,000 population in 5 years period from 2015 to 2019 when compared to the research results obtained in Thailand in 2010 (33.36 cases per 1,000 population in 2010 and average 33.79 cases per 1,000 population/year from 2015 to 2019). There were many factors that affected the lower incidence of diarrheal disease. In addition to WASH intervention from UCH, quality improvements in socioeconomic status, access to ORS, and the implementation of a rotavirus vaccine (RV) as an optional vaccine in 2012 are possible.

The majority of IPD patients were infants (children under 1 year of age) and toddlers children aged between 1 and less than 2 years), there were 57.3 case per 1,000 person-years (30.2%), and 51.6 case per 1,000 person-years (29.5%), respectively. Similar to what was seen in previous studies in different periods in both Thailand and other countries. [5, 10, 11, 14, 19] A published investigation from Thailand over a 1-year period from 1985 to 1986 [20] studied children under 5 years of age with acute diarrhea. Half of them were younger than 1-year-old, and 84% were younger than 2 years old. In 2006, Wilunda et al. [21] studied factors associated with diarrhea in Thai children under 5 years of age in 2006 and concluded that the age group between 6 and 23 months of age was the high-risk group for diarrheal disease. Consistent with studies in Asia (Federal Democratic Republic of Nepal) and Africa (Arab Republic of *Egypt* and Federal Democratic Republic of *Ethiopia*), this age group is seen to have a high risk of diarrhea, which may be affected by immature intestinal immunity to enteropathogens and environmental exposure, and may be related to the introduction of complementary food [10, 22, 23]. The most common cause relating to admission was form category A09: other gastroenteritis and colitis of infectious and unspecified origin; (83.0%). This causative category was the same as in 2010 [5].

Along with the diarrheal-related mortality rate in our country, it continuously declined from 2.0 to 0.7 per 100,000 population in 1978 to 1983 [19] and sharply decreased by 70.8% (-82.4 to -51.0%) from 2005 to 2015. On a broader scale, it decreased by 39.2% among children under 5 years of age in this period [6]. In 2010, diarrhea-related death rate was 1.36 per 100,000 population per year [5]. As in our study, the death rate from diarrhea remained constant at a lower rate from 2010, which is approximately 31 to 47 deaths/year or 0.71 to 1.16 per 100,000 population per year in the 5-year study period. The diarrhea-related death rate was highest in the first year of life, which was attributed to 64.9% (126/194) of all deaths or 4.1 deaths per 100,000 person-years (*P* < 0.001). The highest mortality cause was associated with A09: other gastroenteritis and colitis of infectious and unspecified origin, which comprised 81.4–96.8% of all deaths, the same as the highest cause of diarrhea-related admission. In Thailand, there are three factors that contributed to the lower rate of diarrheal deaths, similar to the factors that affect the lower incidence rate, of which the first is the achievement of national insurance, especially the UC scheme in 2002, that covers nearly the entire Thai population. Second, there are national and accessible oral rehydration therapies, or ORS, and improvements in WASH interventions. Last, the implementation of RV as an optional vaccine in 2012. All these factors may have had a positive impact on the incidence and mortality rate among Thai children. Notably, in our study, an approximately two thirds of diarrheal-related mortality rate occurred in children under 1 year of age, which comprised 126/194 deaths or 4.1 deaths per 100,000 person-years in the 5-year period (*P* < 0.001). Similar to a previous study published in 2010, the highest diarrheal-related mortality occurred in children in their first year of life, with 34 deaths per 100,000 population. Both diarrheal-related admission and mortality rates were highest in first year of life, then gradually declined with increasing age. The reasons be related to the immature of the immune system and personal hygiene [10, 22, 23].

Nearly total of diarrheal-related admission cases had LOS less than 14 days, meaning that the diarrhea was acute and self-limited within this period and had not turned into chronic form. Moreover, the diarrheal-related mortality rate in LOS less than 14 days group was small compared to the group with an LOS of more than 14 days. In patients whose LOS was longer than 14 days, which turned to chronic diarrhea, these patients might have had co-morbidity or underlying diseases such as malnutrition, infection, or disorder of fluid and acid–base balance as described in previous study [5]. The total medical expenditure for management of acute diarrhea-related admission in Thailand in 2010 was 905,784,298 Thai baht, or 30,035,807 USD (December 31, 2010, US Federal Statistical Release year) [14]. For children under 5 years of age, the hospital charge was 351,340,573 Thai baht, or 11,711,352 USD [5]. The cost of hospital-based patients with acute diarrhea in children under 5 years was as high as one third of total cost. For our study, the estimated hospital expenditure was higher than in the past decade even though the incidence of acute diarrhea was lower (443,782,932 Thai baht/year or 13,266,656.7 USD/year). The highest healthcare expenditure was found in 2018 as the highest incidence of diarrheal-related admission that was found in our study. That might indicate that acute diarrhea is still the burden of our country.

The constant pattern of seasonal variation demonstrated consistency over the 5-year period. The higher peak in winter (November to February) was followed by a smaller peak in the early rainy season (May to July), similar to prior data obtained in 2010 [5]. The peak during the cool weather period might be from rotavirus outbreaks in dry and cool conditions in which microorganisms tend to peak, also called winter diarrhea. Previous literature reviewed the seasonal effect on the incidence of acute diarrhea in Northeast Thailand from 1982 to 1987 and reported that the apex of the incidence was inversely related to an abrupt decrease in the temperature in the first month of each year [24]. While the minor peak could be from dysentery or rainy-season diarrhea, this bacterial diarrhea peaked in hot, dry, and early rainy climates. [5, 12, 19, 25–29]. The peak winter diarrhea decreased with age, while the rainy-season diarrheal peak increased with advanced age [24]. In contrast with a study from the Federal Democratic Republic of Nepal [22], their results indicated a unique seasonal difference, which was a risk of incidence of diarrheal-related admission in spring and summer and a lower risk of disease in autumn and winter. They hypothesized that it was caused by bacteria because the peaks usually occurred in hotter and rainier months [30]. This may be the result of different topographies and climates determining the effect of spreading pathogens.

The high density of patients in each geographical area with Cholera, Typhoid, and Amoebiasis may be explained by the environment being suited for the growth of pathogens. For Cholera, which causes severe watery diarrhea, had a high prevalence in the Bangkok metropolis of central and Chonburi Province in the eastern region. These industrial cities are overcrowded with various nationalities and tourists, and therefore easily transmit fecal–oral pathogens. Chonburi Province is a coastal area with brackish water, which is a great habitat for *Vibrio* *cholera.* For Typhoid, this pathogen was highest in the southernmost part of the country (Narathiwat, Songkhla, and Pattani provinces), these coastal provinces were not only crowded by tourism but also rich in seafood, which was a great source of typhoidal and nontyphoidal species. Lastly, Amoebiasis also occurred in the southern region where is a good environment of this organism. This geographical distribution was as same as surveillance of diarrheal disease in Thailand during the period 1987 to 1973 [19].

Due to the limitation that current practice does not recommend identifying all causative pathogens causing acute diarrhea in all patients [12], our data were obtained from the ICD-10 TM, and the diagnosis was based on clinical presentation. Nearly all of the hospitalized diarrhea patients were due to A08: viral and other specified intestinal infections; and A09: other gastroenteritis and colitis of infectious and unspecified origin. In Thailand, data from other studies revealed that rotavirus was the major infectious agent causing watery diarrhea and leading to death in children younger than 5 years [31–33]. Rotavirus vaccines have been licensed as optional vaccines in Thailand since 2012 and incorporated in the Expanded Program of Immunization (EPI) in 2020. The effectiveness of RV against acute diarrhea in children under 5 years of age before and after incorporation of RV into the EPI is warranted. However, our study has some limitations. First, this study was retrospective; consequently, the data and results may be inaccurate due to incomplete medical records or ICD-10 TM codes. Moreover, the current practical management and clinical practice guidelines for acute gastroenteritis do not recommend identifying the pathogens in all patients, which might lead to miscoding or misclassification.

## Conclusions

The incidence rate of acute diarrhea in Thai children under five years of age was higher while the mortality rate of acute diarrhea was lower than those in the past decade, but the cost of treatment is still high. The two most common cause contributing to admission A08: viral and other specified intestinal infections and A09: other gastroenteritis and colitis of infectious and unspecified origin. A similar seasonal outbreak of acute diarrhea was observed each year. The causative agent was not significant and was mainly unspecific.

## Data Availability

The data that support the findings of this study are available from Thailand National Health Coverage but restrictions apply to the availability of these data, which were used under license for the current study, and so are not publicly available. Data are however available from the authors upon reasonable request and with permission of Thailand National Health Coverage.

## Abbreviations

NHC: National Health Coverage
ICD-10-TM: International Statistical Classification of Diseases and Related Health Problems, tenth Revision, Thai Modification
WHO: World Health Organization
UNICEF: United Nations Children's Fund
GBD: Global Burden of Diseases, Injuries, and Risk factor Study
ORS: Oral rehydration salts
UC: Universal Coverage Scheme
CSMBS: Civil Servant Medical Benefit Scheme
LOS: Length of hospital stay
IPD: In-patient department
USD: United States dollar
RV: Rotavirus vaccine
EPI: Expanded Program of Immunization
